# Autophagy Activation Is Involved in 3,4-Methylenedioxymethamphetamine (‘Ecstasy’)—Induced Neurotoxicity in Cultured Cortical Neurons

**DOI:** 10.1371/journal.pone.0116565

**Published:** 2014-12-31

**Authors:** I-Hsun Li, Kuo-Hsing Ma, Shao-Ju Weng, Shiang-Suo Huang, Chang-Min Liang, Yuahn-Sieh Huang

**Affiliations:** 1 Department of Pharmacy Practice, Tri-Service General Hospital, National Defense Medical Center, Taipei, Taiwan; 2 Department of Biology and Anatomy, National Defense Medical Center, Taipei, Taiwan; 3 Department of Pharmacology and Institute of Medicine, College of Medicine, Chung Shan Medical University, Taichung, Taiwan; 4 Department of Ophthalmology, Tri-Service General Hospital, National Defense Medical Center, Taipei, Taiwan; 5 Graduate Institute of Aerospace and Undersea Medicine, National Defense Medical Center, Taipei, Taiwan; Children's Hospital of Pittsburgh, University of Pittsburgh Medical Center, United States of America

## Abstract

Autophagic (type II) cell death, characterized by the massive accumulation of autophagic vacuoles in the cytoplasm of cells, has been suggested to play pathogenetic roles in cerebral ischemia, brain trauma, and neurodegenerative disorders. 3,4-Methylenedioxymethamphetamine (MDMA or ecstasy) is an illicit drug causing long-term neurotoxicity in the brain. Apoptotic (type I) and necrotic (type III) cell death have been implicated in MDMA-induced neurotoxicity, while the role of autophagy in MDMA-elicited neurotoxicity has not been investigated. The present study aimed to evaluate the occurrence and contribution of autophagy to neurotoxicity in cultured rat cortical neurons challenged with MDMA. Autophagy activation was monitored by expression of microtubule-associated protein 1 light chain 3 (LC3; an autophagic marker) using immunofluorescence and western blot analysis. Here, we demonstrate that MDMA exposure induced monodansylcadaverine (MDC)- and LC3B-densely stained autophagosome formation and increased conversion of LC3B-I to LC3B-II, coinciding with the neurodegenerative phase of MDMA challenge. Autophagy inhibitor 3-methyladenine (3-MA) pretreatment significantly attenuated MDMA-induced autophagosome accumulation, LC3B-II expression, and ameliorated MDMA-triggered neurite damage and neuronal death. In contrast, enhanced autophagy flux by rapamycin or impaired autophagosome clearance by bafilomycin A1 led to more autophagosome accumulation in neurons and aggravated neurite degeneration, indicating that excessive autophagosome accumulation contributes to MDMA-induced neurotoxicity. Furthermore, MDMA induced phosphorylation of AMP-activated protein kinase (AMPK) and its downstream unc-51-like kinase 1 (ULK1), suggesting the AMPK/ULK1 signaling pathway might be involved in MDMA-induced autophagy activation.

## Introduction

Macroautophagy (henceforth referred to as autophagy) is a highly conserved cellular catabolic process whereby organelles and soluble and aggregated cellular components are enveloped in double-membrane vesicles called autophagosomes, which eventually fuse with lysosomes, leading to the degradation and reuse of the vesicular contents [1]–[5]. Autophagy occurs constitutively at a basal level in all eukaryotic cells and operates as a homeostatic mechanism [6]. In this role, autophagy removes unwanted cellular structures by the degradation of excess or damaged organelles and proteins and thereby contributes to the routine turnover of cytoplasmic components [7]. In addition, autophagy can be activated in response to various cellular and environmental stress conditions (e.g., starvation, oxidative stress) to promote cell survival, or to act as a mode of cell death (e.g., cerebral ischemia) [4], [8], [9]. Autophagic cell death (also called type II programmed cell death) is characterized by the massive accumulation of autophagic vacuoles in the cytoplasm of cells as they die [9], [10]. Defective autophagy has been connected to many human diseases including cancer, myopathies, and neurodegenerative diseases such as Alzheimer's disease, Parkinson's disease, Huntington's disease, and amyotrophic lateral sclerosis [5], [11], [12].

Autophagy involves several steps and is regulated by a large number of autophagy-related genes (Atg) that are conserved from yeast to humans [13]; so far more than 30 have been found. The steps include (i) induction, which involves formation of the phagophore, a double-membrane structure, and is largely dependent on Atg1/unc-51-like kinase 1 (ULK1) complex activation mediated by AMP-activated protein kinase (AMPK) and other factors that lead to the dephosphorylation of mammalian target of rapamycin (mTOR) [14]; (ii) nucleation, which is driven by Vps34, a class III phosphatidylinositol 3-kinase (PI3K), complexed with beclin-1 (Atg6) [4]; (iii) elongation, which is a critical step in the formation of complete autophagosomes and is controlled by two ubiquitin-like conjugation systems (Atg12-Atg5 and Atg8 [microtubule-associated protein 1 light chain 3 {LC3}]-phosphatidylethanolamine conjugated to the nascent autophagosome membrane) [7]; and finally (iv) maturation and degradation, which involve fusion with lysosomes to form autolysosomes, and degradation of the luminal contents [7], [10], [15].

3, 4-Methylenedioxymethamphetamine (MDMA or Ecstasy) is one of the most popular recreational drugs abused by adolescents [16]. Accumulating evidence indicates that long-term MDMA abuse is associated with cognitive impairments and mood disturbances [17], [18]. In the central nervous system (CNS), MDMA is toxic to both serotonergic neurons and the dopaminergic system [19], [20]. In addition, MDMA is toxic to brain regions, including the cerebral cortex, thalamus, and striatum [18], [21]. Oxidative stress, excitotoxicity, mitochondrial dysfunction, and necrosis have been implicated in MDMA-induced neurotoxicity [22]. MDMA also induces apoptosis (type I programmed cell death) by increasing the expression of the pro-apoptotic protein Bax and inhibition of the anti-apoptotic protein Bcl-2 [23]. A previous study reported that the transcript expression level of Atg5 is elevated in mouse embryo and neuroblastoma cells after MDMA treatment [24]. However, the pathophysiological role of autophagy in MDMA-induced neurotoxicity is unknown. The aim of the present study is to investigate the effect of MDMA on autophagy in cultured rat cortical neurons. In this study, MDMA treatment provoked a dramatic increase in autophagy activation in cortical neurons, neurite degeneration, and neuronal death rate. Inhibition of autophagy with 3-methyladenine (3-MA) significantly decreased autophagosome accumulation and prevented neurite damage and neuronal death. Our data suggest that excessive autophagy is a contributing factor in MDMA-induced neurotoxicity. Furthermore, AMPK/ULK1 signaling might be involved in the MDMA-induced autophagy pathway.

## Materials and Methods

### Antibodies and reagents

The following antibodies were used: rabbit anti-LC3B, mouse anti-MAP2, and FITC-conjugated goat anti-rabbit IgG (Sigma Chemical, St. Louis, MO); rabbit anti-beclin-1 and mouse anti-β-actin (Santa Cruz Biotechnology, Santa Cruz, CA); mouse anti-NeuN (Millipore, Temecula, CA); rabbit anti-phospho-AMPK (Thr172), rabbit anti-AMPK, rabbit anti-phospho-mTOR (Ser2448), rabbit anti-mTOR, rabbit anti-phospho-ULK1 (Ser555), and rabbit anti-ULK1, rabbit anti-cleaved caspase 3, rabbit anti-caspase 3 (Cell Signaling, Danvers, MA); Texas red-conjugated goat anti-mouse IgG (Vector Laboratories, Burlingame, CA); Horseradish peroxidase (HRP)-conjugated anti-rabbit and anti-mouse immunoglobulin (Jackson ImmunoResearch Laboratories, West Grove, PA). 3-Methyladenine, rapamycin, bafilomycin A1, and monodansylcadaverine (MDC), wortmannin, LY294002 (Sigma Chemical) and Torin-1 (Tocris Bioscience, Ellisville, MO) were used in our autophagy flux studies. MDMA (purity, 98%) was obtained from the Investigation Bureau of Taiwan.

### Primary cortical neuron culture

All experimental procedures were approved by the Institutional Animal Care and Use Committee at the National Defense Medical Center (Taipei, Taiwan). Rat primary cortical neuron culture was performed according to a published procedure with modifications [25]. One-day-old Sprague–Dawley rats were sacrificed and their brains were removed. Meninges and blood vessels were carefully removed. The cerebral cortex was placed in ice-cold calcium and magnesium-free Hank's balanced saline solution (HBSS, Invitrogen, Carlsbad, CA), minced, digested with 0.125% trypsin and 0.05% DNase for 10 min at 37°C, suspended in DMEM with 10% fetal bovine serum (FBS) to inactivate the trypsin, and pelleted by centrifugation at 1000 rpm for 10 min. After suspending the cell pellet in medium and adjusting the cell density to 1×10^6^ cells/ml, neurons were seeded on coverslips or culture dishes precoated with poly-D-lysine and cultured with growth medium (1∶1 mixture of DMEM/F-12 and neurobasal medium [Invitrogen], 5% fetal bovine serum, supplemented with 0.5 mM L-glutamine, 1% penicillin-streptomycin and 2% B-27 serum-free supplement [Invitrogen]). On the third day post-plating, non-neuronal cell proliferation was suppressed by treatment with 5 µM cytosine-D-arabinofuranoside for 24 h. On the fifth day of culture, the cells were used for the experiment. Neurons were identified by positive staining with neuron-specific MAP2 antibody and the percentage of MAP2-immunostained neurons was over 90%.

### Drug treatment

Cells were pretreated with 3-MA (1 mM ), rapamycin (100 nM), bafilomycin A1 (1 nM), Torin-1 (100 nM), wortmannin (5 µM), or LY294002 (5 µM) for 30 min prior to the treatment with MDMA for 24 h or 48 h.

### MTT assay

The MTT assay was performed according to the manufacturer's protocol (Sigma). Cells were treated for 48 h with increasing concentrations of MDMA in quadruplicate for each condition, and they were subsequently incubated with the MTT solution (0.5 mg/ml) at 37°C for another 2 h. Supernatants were then discarded and 200 µl of acidified isopropanol (0.04 N HC1 in isopropanol) were added to the cultures and mixed thoroughly to dissolve the dark blue crystals of formazan. The optical density at 570 nm was measured using a microplate reader. The cell viability was expressed as a percentage of control.

### Monodansylcadaverine staining

The monodansylcadaverine (MDC) assay to label autophagosomes has been described previously [26]. Cells grown on coverslips were treated with MDMA in the presence or absence of 3-MA (1 mM) for 48 h, incubated with 0.05 mM MDC for 30 minutes at 37°C, washed three times with PBS, and mounted on slides. MDC-labeled vacuoles were immediately imaged using a fluorescence microscope (excitation wavelength 380 nm, emission filter 525 nm). The number of cells with MDC-labeled vacuoles were counted in eight randomly chosen field per dish and more than 20 cells in each field are analyzed.

### Immunofluorescence

For double immunofluorescence, cells were fixed in methanol for 5 min, washed 3 times in PBS (5 min for each), simultaneously incubated with rabbit anti-LC3B antibody and mouse anti-MAP2 antibody for 1 h, treated with FITC-conjugated goat anti-rabbit IgG and Texas red-conjugated goat anti-mouse IgG at room temperature for 1 h, washed with PBS, mounted with 3% n-propyl gallate and 50% glycerol in PBS, and viewed under a fluorescence microscope (Nikon, Japan). For NeuN or cleaved caspase 3 immunostaining, cells were fixed in 10% formalin, blocking with 5% non-fat milk in 0.1% Triton X-100, and then incubated with anti-NeuN or cleaved caspase 3 antibody for 1 h. The cells were then incubated with Texas red-conjugated goat anti-mouse IgG or Texas red-conjugated goat anti-rabbit IgG for 1 h and mounted. The number of NeuN-positive or cleaved caspase 3-positive cells was counted in six-eight random fields (10X) on each coverslip (two per condition), and the average number of NeuN-positive cells or cleaved caspase 3 per random field was determined for each condition tested and compared to control group.

### Neurite Outgrowth Assay

Neurite outgrowth visible in MAP2-positive neuron images was quantified as the total length of neurites radiated from a single neuron. Images were acquired from randomly selected fields (n = 8∼12) under fluorescence Microscope. The length of the total neurite of 40–50 neurons per condition was determined using NeuronJ/ImageJ software (version 1.46r; NIH, Bethesda, MD). NeuronJ is an ImageJ plugin to facilitate the tracing and quantification of elongated and network image structures, in particular neurites in fluorescence microscopy images [27]. NeuroJ allows the user to interactively analyze running averages and statistic of neurite length, soma number, neurite attachment points, and neurite ending points from a single image. Each experimental condition was done in duplicate wells, and at least three independent experiments were conducted to acquire the final results. Approximately a total of 200–300 cells were counted in each group.

### Western blot analysis

Cells were washed with cold phosphate-buffered saline (PBS), extracted with RIPA buffer (50 mM Tris, pH 7.4, 150 mM NaCl, 0.1% SDS, 1% NP-40, 1 mM EDTA, 0.5% sodium deoxycholate) containing 1 mM PMSF, 2 mM Na_3_VO_4_, 5 mM NaF, and protease inhibitor cocktail, scraped, lysed by sonication, and centrifuged at 14,000 *g* for 10 min at 4°C. The supernatant protein concentration was determined using a bicinchoninic acid (BCA) protein assay kit (Bio-Rad, Hercules, CA). The cellular homogen (30 µg per lane) was separated by 8–15% SDS polyacrylamide gel electrophoresis and transferred onto a PVDF membrane (Millipore). The membrane was incubated in blocking solution (5% non-fat milk in TBS-T) at room temperature for 1 h, incubated overnight with primary antibody at 4°C, washed with TBS-T, treated with HRP-conjugated secondary antibodies at room temperature for 1 h, washed with TBS-T, and incubated with enhanced chemiluminescence reagents (ECL, Thermo Scientific, Rockford, IL) to visualize the bands. Each experiment was repeated at least 3 times. Densitometric analysis of the immunoreactive bands was performed using ImageJ software (NIH).

### Lactate dehydrogenase (LDH) assay

As an index of cell death, release into extracellular medium was measured using the LDH assay. Briefly, supernatants were collected at the times indicated and intact cells were lysed using Triton X-100-containing lysis buffer. The amount of LDH release was determined spectrophotometrically at 492 nm using the LDH-Cytotoxicity Assay Kit (Sigma). Percent cell death was calculated using the formula: % cytotoxicity  =  LDH release (OD_492_)/maximum LDH release (OD_492_), after correcting for baseline absorbance of the LDH release at 492 nm.

### Statistical analysis

The quantitative data are presented as mean ± S.D. The Student *t* test was used to calculate *P* values, with a *P* value <0.05 defined as statistically significant and a *P*<0.01 as highly statistically significant.

## Results

### MDMA decreased neuronal cell viability in a dose-dependent manner

**Figure 1:**
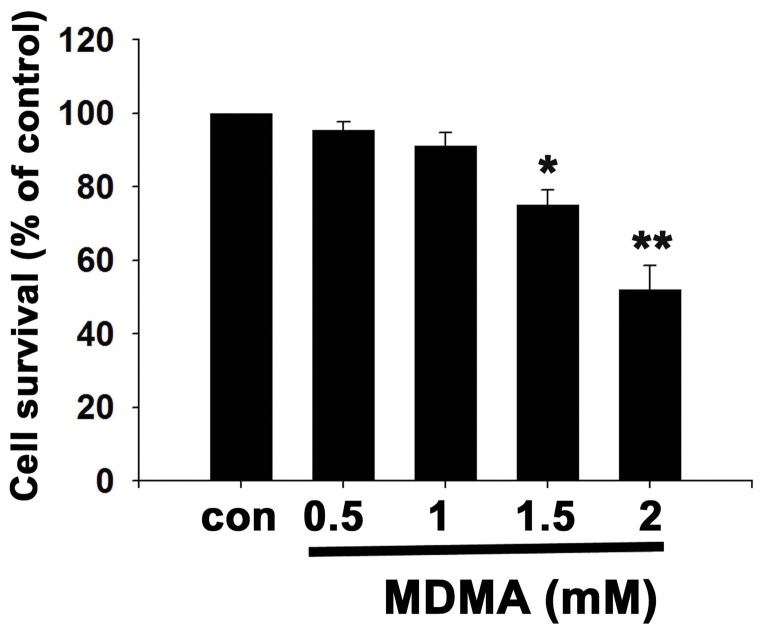
Effect of MDMA on cortical neuron viability. Cultured cortical neurons were treated with different concentration of MDMA for 48 h, and then cell viability was determined by MTT assay. The data are expressed as percentage of untreated controls (mean ± S.D., n = 3, quadruplicate wells for each condition). *P<0.05; **P<0.01 vs control.

We first examined the dose effect of MDMA on cell viability. Cortical neuron cultures were treated with different concentration of MDMA (0.5, 1, 1.5, 2 mM) for 48 h, and then the cell viability was determined by MTT assay. MDMA produced a concentration-dependent decrease in cell viability (Fig. 1). Treatment with 0.5 and 1 mM MDMA for 48 h did not have an obvious effect on the cell viability, as approximately 95% and 91% of the cell viability compared to normal control. When cells were treated with higher concentration of MMDA (1.5 and 2 mM), the cell viability was significantly decreased to 75% and 52%, respectively.

### MDMA induced autophagy and neurite degeneration in a dose- and time-dependent manner

**Figure 2:**
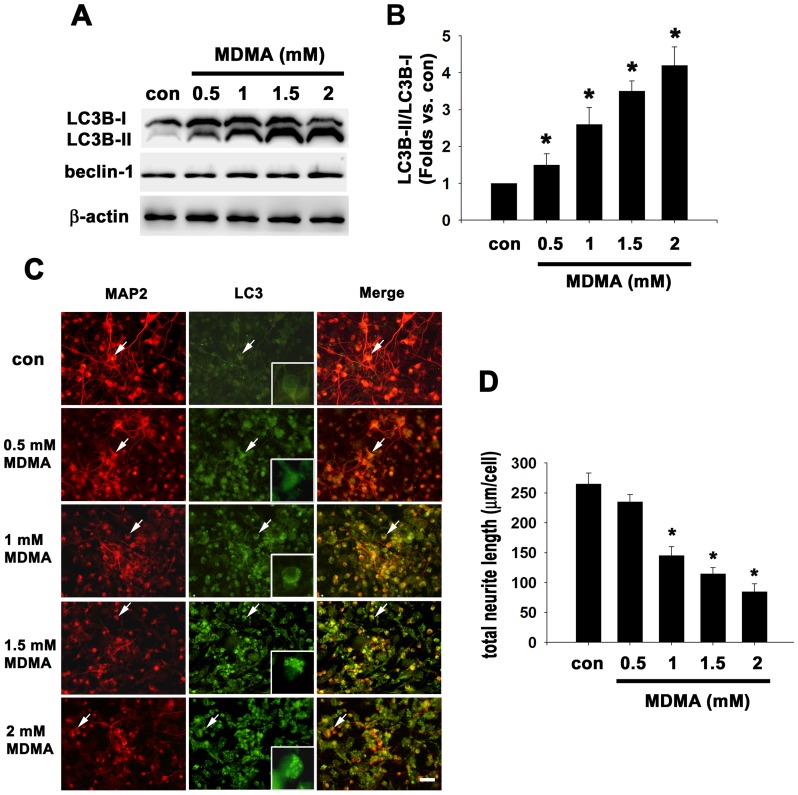
Dose dependence of MDMA-induced autophagy activation and neurite degeneration in cortical neuron culture. Cultured cortical neurons were treated with the indicated concentrations of MDMA for 48 h, and then western blot analysis and double immunofluorescence were performed. (A) Western blot analysis of LC3B and beclin-1. β-actin was used as the internal control. (B) Densitometry of LC3B-II/LC3B-I ratio. Quantitative data are expressed as intensity relative to the control mean value (mean ± S.D.). n = 3, *P<0.05 vs control. (C) Double immunofluorescence stained images and merged images of representative cells using anti-LC3B and anti-MAP2. Bar  = 30 µm. Intense LC3 punctate fluorescence shown in the MDMA-treated group (see insets). (D) Quantitation of neurite outgrowth. The total neurite length are shown as mean ± S.D., n = 3 experiments with 200–300 cells per experiment, *P<0.05 vs control.

We investigated the dose-dependent effect of MDMA on autophagy activity and neurite outgrowth in cultured rat cortical neurons. Autophagy activity was assessed using immunoblotting and immunofluorescence to detect the essential autophagy protein LC3. Upon autophagy activation, the LC3-I protein localized in the cytoplasm is cleaved, lipidated, and inserted as LC3-II into autophagosome membranes. Thus, an increase in the amount of the smaller-molecular-weight LC3-II protein and an increase in the LC3-II/LC3-I ratio are a hallmark of autophagy and correlate with an increased number of autophagosomes [28]. In the present study, cortical neurons were treated with different concentrations (0.5 mM, 1 mM, 1.5 mM, 2 mM) of MDMA for 48 h and then performed western blot analysis using LC3 antibody. The results revealed a dose-dependent increase in ratios of LC3B-II/LC3B-I. Treatment with MDMA at 0.5, 1, 1.5, 2 mM significantly increased the ratios of LC3B-II/LC3B-I to 1.5-fold, 2.6-fold, 3.5-fold, and 4.2-fold of the control values, respectively (n = 3, *p*<0.05) (Fig. 2A and 2B). Increase in ratio of LC3B-II/LC3B-I are known to represent upregulation of autophagy. Beclin-1 expression are known to be involved in the formation of preautophagosomal structures. However, the expression level of beclin-1 was not affected by MDMA (Fig. 2A). We then performed double immunofluorescence to correlate the LC3 expression and neurite outgrowth using anti-MAP2 (a general neurite marker) and anti-LC3B antibodies after 48 h of MDMA treatment. As shown in Fig. 2C, in non-treated control neurons, the pattern of LC3B immunoreactivity was light, diffuse, and cytoplasmic within the cell body. At 500 µM of MDMA treatment, anti-LC3B staining exhibited a more condensed diffuse distribution. After exposure to 1 mM MDMA, typical cytoplasmic LC3B punctate was formed notably in almost all neurons with shortening of total neurite length per neuron from 265±18 µm (control group) to 145±15 µm (1 mM MDMA treatment). At higher concentrations of MDMA (1.5 and 2 mM), more LC3 punctate spots were observed and accompanied by dose-dependent decreases in neurite length per neuron to 115±10 µm and 85±13 µm, respectively (n = 3 experiments with 200–300 cells per experiment) (Fig. 2C and 2D). Immunofluorescence demonstrated that MDMA triggers dose-dependent autophagosome formation accompanied by reduction of neurite outgrowth in cultured neuronal cells (also see inset showing a magnified view of LC3B-labeled aggregation in MDMA-treated neuron in Fig. 2C).

**Figure 3:**
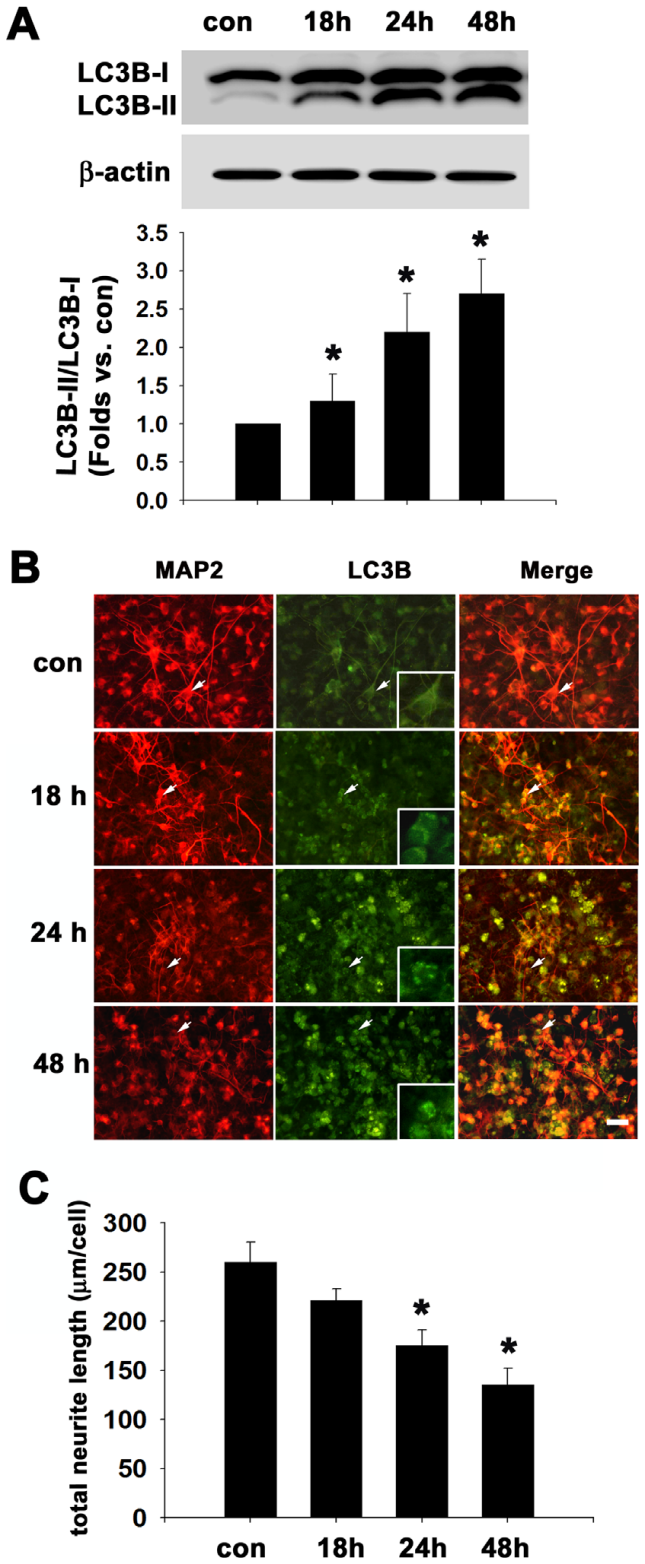
The time course of MDMA-induced autophagy activation in cultured cortical neurons. Cultured cortical neurons were treated with MDMA for different times as indicated, and then subjected to western blot analysis and double immunofluorescence staining with anti-LC3B and MAP2 antibodies. (A) Western blot analysis of LC3B. β-actin was used as the internal control (upper panel). Densitometry of LC3B-II/LC3B-I (bottom panel). Quantitative data are expressed as intensity relative to the control mean value (mean ± S.D.). n = 3, *P<0.05 vs control. (B) Representative double immunofluorescence and merged images using anti-LC3B and anti-MAP2 antibodies. Bar  = 30 µm. Intense LC3 punctate staining pattern shown in the MDMA-treated group (see insets). (C) Quantitation of neurite outgrowth. The total neurite length are shown as mean ± S.D., n = 3 experiments with 200–300 cells per experiment, *P<0.05 vs control.

Although 1 mM MDMA had no obvious effect on cell viability, pharmacological actions significantly caused a decrease in neurite outgrowth and upregulation of autophagy. Therefore, 1 mM MDMA was applied to cultures in the following experiments. We then examined the time dependent effect of MDMA on autophagy activation. The cells were treated with 1 mM MDMA for 18 h, 24 h or 48 h and subjected to western blot analysis using LC3 antibody. The results showed that treatment with MDMA for 18, 24 and 48 h significantly induced a time-dependent increase in ration of LC3B-II to LC3B-I to 1.3-fold, 2.2-fold, and 2.7-fold of control values, respectively (n = 3, *p*<0.05) (Fig. 3A). Double immunofluorescence staining with anti-MAP2 and anti-LC3B antibodies appeared that LC3B punctate staining was not observed within 13 h of MDMA treatment (data not shown), whereas positive aggregations increased rapidly within 18 h and even more punctate staining was observed after 24 h and 48 h of MDMA treatment (see inset showing a magnified view of LC3B-labeled neuronal cells in Fig. 3B). Moreover, the neurite outgrowth was significantly reduced by 32% and 48% following MDMA treatment for 24 and 48 h, respectively, when compared with control cells (n = 3 experiments with 200–300 cells per experiment) (Fig. 3C). MDMA removal for 48 h reduced autophagosome accumulation almost to the baseline, suggesting that this process is reversible (data not shown).

### Autophagy inhibitor, 3-MA decreases MDMA-induced activation of autophagy, and protects neurons against neurite degeneration and neuronal death

**Figure 4:**
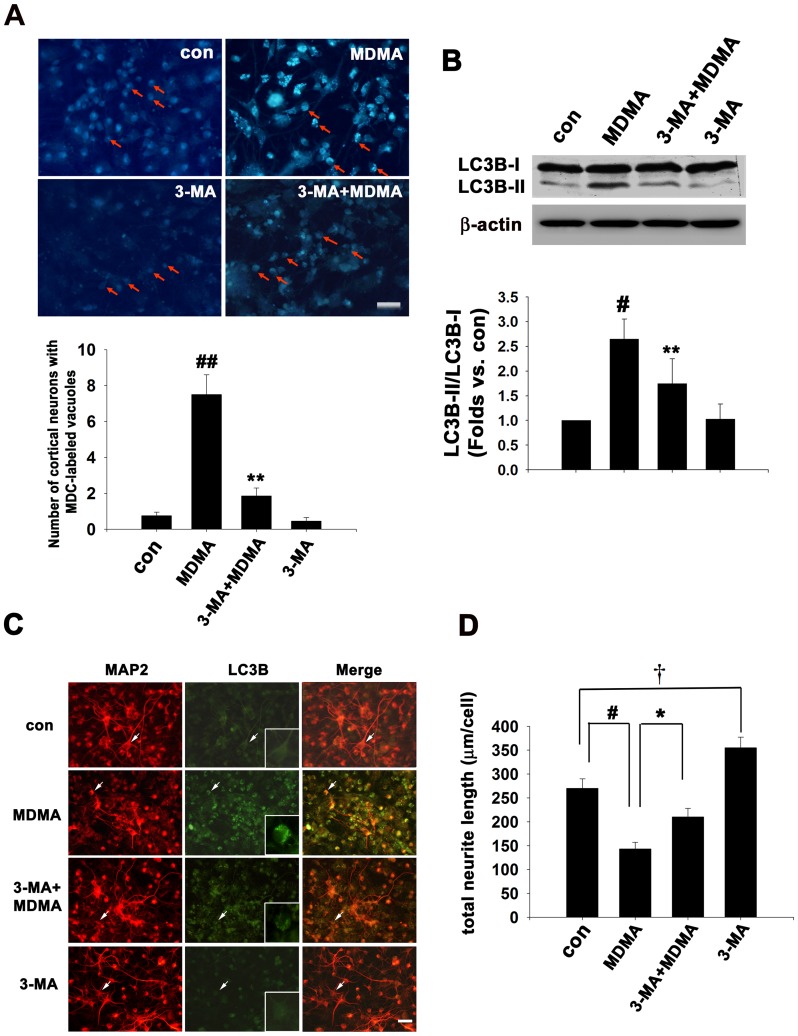
3-MA decreased MDMA-elicited autophagy activation and neurite degeneration. Cultured cortical neurons were exposed to 1 mM MDMA for 48 h in the presence or absence of 1 mM 3-MA for 48 h. Autophagy activity was assay by MDC incorporation, western blot analysis and double immunofluorescence staining with anti-LC3B and MAP2 antibodies. (A) The autophagic vacuoles were visualized by monodansylcadaverine (MDC) staining and the mean number of MDC-labeled vacuoles per cortical neurons (arrows) was quantitation. Quantitative data are expressed as mean ± S.D. n = 3. ^##^ *P*<0.01 vs control, **P<0.01 vs MDMA-treated group. Bar  = 30 µm. (B) Western blot analysis of LC3B expression. β-actin was used as the internal control. Immunoblot signal ratio of LC3B-II-to-LC3B-I was quantitative by densitometric assay. Quantitative data are expressed as intensity relative to the control mean value (mean ± S.D.). n = 3. ^#^ *P*<0.05 vs control, **P<0.01 vs MDMA-treated group. (C) Representative double immunofluorescence and merged images using anti-LC3B and anti-MAP2 antibodies. Bar  = 30 µm. (D) Quantitation of neurite outgrowth. The total neurite length are shown as mean ± S.D. n = 3 experiments with 200–300 cells per experiment, ^#^ *P*<0.05 vs control; *P<0.05 vs. MDMA treatment; **^†^** *P*<0.05 vs control.

**Figure 5:**
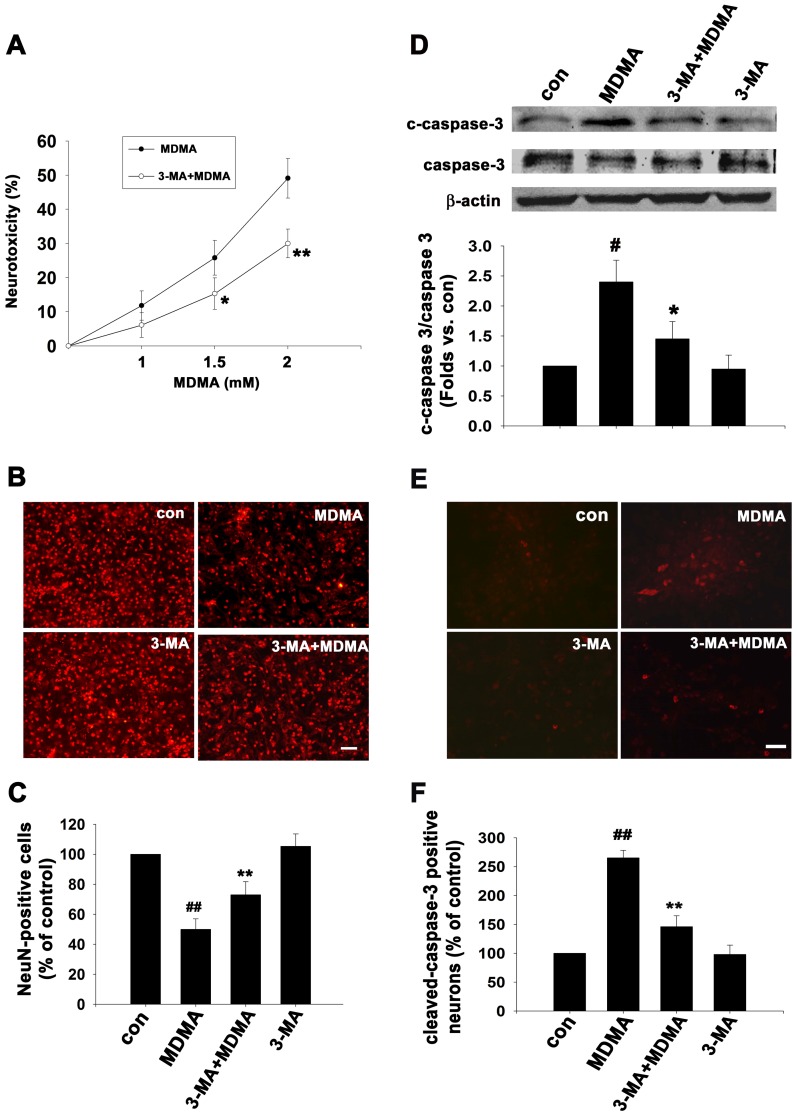
3-MA alleviated MDMA-induced cortical neuron death. (A) Cortical neuron cultures were treated with different concentrations of MDMA in the presence or absence of 1 mM 3-MA. After 48 h, the neuronal death was determined by LDH cytotoxicity assay. The data are presented as percentage of dead cells. n = 3, triplicate wells for each condition, *p<0.05, **p<0.01 vs control. (B) Cortical neuron cultures were treated with 2 mM MDMA in the presence or absence of 1 mM 3-MA. After 48 h, the cells were immunostained with anti-NeuN antibody. Bar  = 50 µm. (C) Quantitative data of the number of NeuN-positive cells. The data are presented as percentage of the control value (mean ± S.D.). n = 3, ^##^ *P*<0.01 vs control; **p<0.01 vs 2 mM MDMA treatment. (D) 3-MA attenuated MDMA-induced activation of caspase 3. Cortical neuron cultures were treated with 2 mM MDMA in the presence or absence of 1 mM 3-MA for 24 h, and then western blot analysis was performed using antibodies against cleaved-caspase 3 and caspase 3. β-actin was used as the internal control. Quantitative data are expressed as intensity relative to the control mean value (mean ± S.D.). n = 3, ^#^ *P*<0.05 vs control; *p<0.05 vs 2 mM MDMA treatment. (E) Representative images from cortical neuron cultures treated with 2 mM MDMA in the presence or absence of 1 mM 3-MA. After 24 h, the apoptotic cells were determined by immunostaining with anti-cleaved caspase 3. Bar  = 30 µm. (F) Quantitative data of the number of cleaved-caspase 3 positive neurons. The data are presented as percentage of the control value (mean ± S.D.). n = 3, ^##^ *P*<0.01 vs control; **p<0.01 vs 2 mM MDMA treatment.

Monodansylcadaverine (MDC) staining can be used to detect autophagic vacuoles (AVs) [29]. The autofluorescent drug MDC accumulates in AVs, but not in the early endosome compartment. Under a fluorescence microscope, AVs stained by MDC appear as distinct punctuate structures throughout the cytoplasm or within the perinuclear region. As shown in Fig. 4, the number of MDC-labeled vesicles within cortical neurons (arrows in Fig. 4A) was significantly increased to 7.5±1.1 at 48 h after MDMA treatment as compared to control values (0.75±0.2), confirming that MDMA induces accumulation of autophagic vesicles. The autophagy inhibitor 3-MA is commonly used to define the role of autophagy under various physiological conditions [30]. Pretreatment with 1 mM 3-MA effectively decreased the number (1.85±0.45) of MDMA-induced MDC incorporation (Fig. 4A), whereas 3-MA treatment alone had no effect on MDC incorporation. To further address the role of MDMA in autophagy induction, cells were pre-incubated with 3-MA for 30 min prior to induction of autophagy by MDMA for 48 h. Western blot analysis showed that 3-MA significantly prevented MDMA-induced ratio of LC3B-II/LC3B-I from 2.6-fold to 1.7-fold of control values (n = 3, *p*<0.01) (Fig. 4B). Cells were treated as the same above, and then double immunostaining were performed with anti-MAP2 and anti-LC3B. 3-MA significantly reduced LC3 punctate staining (see inset showing a magnified view of LC3B-labeled neuronal cells in Fig. 4C) and significantly prevented nerve fiber degeneration (Fig. 4C and 4D). Interestingly, treatment with 1 mM 3-MA alone significantly promote neurite outgrowth (Fig. 4C and 4D). We advance to prove 3-MA protection against MDMA-induced neuronal cell death. Cells were pre-incubated with 3-MA for 30 min prior to different concentration of MDMA treatment for 48 h, then the neuronal death was determined by LDH assay. As shown in Fig. 5, cell death increased from 11.8% to 49.1% as MDMA concentration increased from 1.0 mM to 2.0 mM, but 3-MA pretreatment decreased 1.0 and 2.0 mM MDMA-induced cell death to 6.1% and 30%, respectively (n = 3, triplicate wells for each condition). Morphological data by anti-NeuN immunostaining also confirmed that when cultured neuron were treated with 2 mM MDMA for 48 h, 3-MA significantly attenuated MDMA-induced neuronal cell loss (n = 3, *p*<0.01) (Fig. 5B and 5C). We further investigated the apoptotic effect of inhibition of autophagy by 3-MA after MDMA exposure. The appearance of cleaved caspase-3 immunoreactivity is used as a marker of apoptosis. The antibody that was used detects endogenous levels of the large fragment (17/19 kDa) of activated caspase-3 resulting from cleavage adjacent to Asp^175^, and does not recognize full length caspase-3 or other cleaved caspases. Cortical neuron cultures were treated with 2 mM MDMA in the presence or absence of 1 mM 3-MA for 24 h, and then western blot analysis and immunofluorescence were performed using antibodies against cleaved-caspase 3 and caspase 3. As shown in Fig. 5D, MDMA significantly caused an increase in cleaved caspase-3 protein level. 3-MA pretreatment significantly attenuated the upregulation of cleaved caspase 3 from 2.4-fold to ∼1.4-fold of control values (n = 3, *p*<0.05) (Fig. 5D). The immunofluorescence data also confirmed that 3-MA pretreatment significantly reduced the percentage of immunopositive cleaved caspase-3 neurons induction by 24 h of MDMA treatment from 265±13% to 146±19% of control values (n = 3, *p*<0.01) (Fig. 5E and 5F), whereas very low number of cleaved-caspase 3 positive cells were detected in untreated control cells or 3-MA treatment alone. Therefore, MDMA-induced neuronal death are at least partly autophagy-dependent. However, other autophagy inhibitors, wortmannin and LY294002, had no effect on MDMA-elicited autophagy and neurite damage (data not shown).

### Autophagosome accumulation is associated with MDMA-induced neurite degeneration

**Figure 6:**
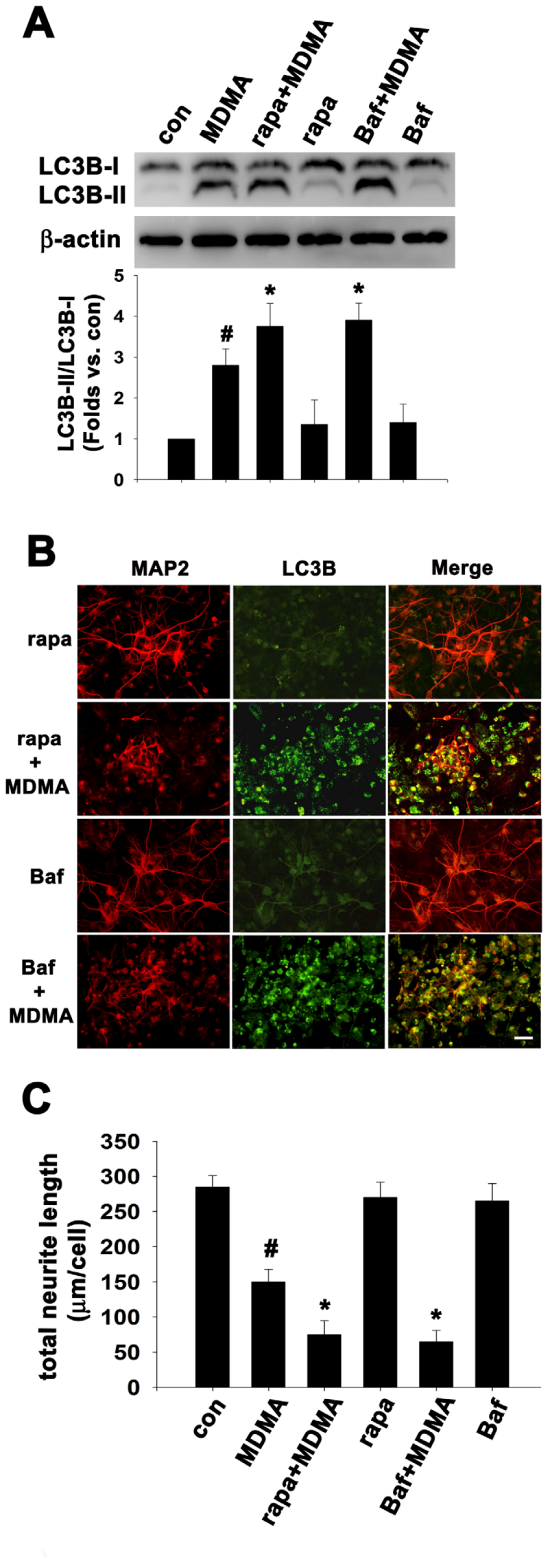
Rapamycin or Bafilomycin augmented MDMA-induced autophagy and neurodegeneration. Cultured cortical neurons were treated with MDMA with/without 100 nM rapamycin (rapa) or 1 nM Bafilomycin A1 (Baf) for 48 h, respectively. After treatment, the cells were performed western blot analysis or double immunofluorescence with anti-LC3B and anti-MAP2 antibodies. (A) Western blot analysis of LC3B expression. β-actin was used as the internal control. Densitometry of LC3B-II/LC3B-I. The data are presented as intensity relative to the control mean value (mean ± S.D.). n = 3, ^#^ *P*<0.05 vs control, *P<0.05 vs MDMA-treated group. (B) Representative double immunofluorescence and merged images using anti-LC3B and anti-MAP2 antibodies. Bar  = 30 µm. (C) Quantitation of neurite outgrowth. The total neurite length are shown as mean ± S.D., n = 3 experiments with 200–300 cells per experiment, ^#^ *P*<0.05 vs. control group; ^*^ *P*<0.05 vs. MDMA treatment.

To further clarify the detrimental role of autophagosome accumulation in MDMA-induced neurotoxicity, we employed rapamycin and Bafilomycin A1. Rapamycin , an mTOR inhibitor commonly used to activate/enhance autophagosome formation. Bafilomycin A1, a specific inhibitor of vacuolar H^+^ ATPase (V-ATPase), prevents autophagy at a late stage by inhibiting fusion between autophagosomes and lysosomes [31], resulting in autophagosome accumulation. Cells were treated with MDMA in the presence or absence of rapamycin or Bafilomycin for 48 h and double immunostained with anti-MAP2 and anti-LC3B antibodies. As shown in Fig. 6, western blot analysis confirmed that cotreatment of MDMA with rapamycin or Bafilomycin A1 synergistically resulted in autophagy upregulation, as determined by an increase in ratio of LC3B-II/LC3B-I from 2.8-fold to 3.7-fold or 3.9-fold of control values, respectively (n = 3, *p*<0.05) (Fig. 6A). 100 nM rapamycin or 1 nM Bafilomycin A1 alone increased LC3B immunostaining intensity slightly but had no effect on neuronal morphology and neurite growth (Fig. 6B and 6C). However, rapamycin and Bafilomycin A1 in the presence of MDMA acted synergistically with MDMA to enhance autophagosome accumulation and neurite degeneration (Fig. 6B and 6C), indicating that autophagosome accumulation is associated with MDMA-induced reduction of neurite outgrowth. Another mTOR inhibitor Torin-1 directly inhibits both mTORC1 and mTORC2. Cotreatment of MDMA with Torin-1 (100 nM) also synergistically resulted in LC3B-labed autophagosome accumulation and aggravated neurite degeneration, whereas Torin-1 alone increased LC3B immunostaining intensity slightly without affecting neurite outgrowth (S1 Fig.). This result was similar to that observed in treatment with rapamycin.

### MDMA induced AMPK/ULK1 signaling

**Figure 7:**
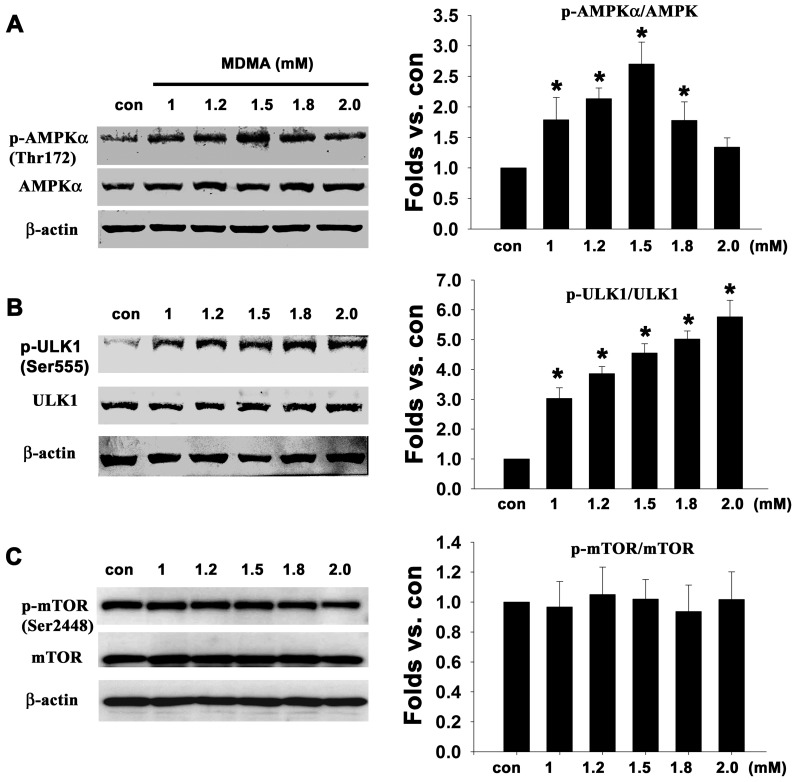
Autophagic signal cascade induced by MDMA. Cultured cortical neurons were treated with the indicated concentrations of MDMA for 48 h, and then western blot analysis was performed using antibodies against phospho-AMPK and AMPK (A), phospho-mTOR and mTOR (B), phospho-ULK1 and ULK1 (C). β-actin was used as the internal control. Quantitative data are expressed as intensity relative to the control mean value (mean ± S.D.). n = 3 experiments, ^*^ *P*<0.05 vs control.

AMP-activated protein kinase (AMPK) is the key signal triggering autophagy through inactivation of mTOR and phosphorylation of ULK1 [32], [33]. AMPK promotes autophagy by directly activating ULK1 through phosphorylation of Ser317, Ser555, and Ser777, while mTOR, another serine/threonine kinase, impedes autophagy by disrupting the interaction between ULK1 and AMPK [14], [32]. Therefore, the deactivation of mTOR triggers autophagy. To further investigate the kinase involved in MDMA-induced autophagy, levels of phosphorylated AMPK, mTOR, and ULK1 were measured. As shown in Fig. 7, MDMA induced an increase in phosphorylation of AMPK in Thr 172, a peak at 1.5 mM MDMA showing a 2.7-fold increase compared to the control cells, and declined after the treatment (Fig. 7A). MDMA also caused a dose-dependent increase in the phosphorylation of ULK1 in Ser 555 (Fig. 7B), whereas did not alter mTOR phosphorylation in Ser 2448 (Fig. 7C), suggesting the possible involvement of AMPK/ULK1, but not mTOR in MDMA-induced autophagy.

## Discussion

MDMA is a neurotoxin. MDMA-induced toxicity may involve Ca^2+^ overload, oxidative stress, and mitochondrial dysfunction, and may lead to apoptotic and necrotic cell death [34]. Autophagy activation involves in brain ischemia, trauma and neurodegeneration. In the present study, we examined the role of autophagy in MDMA-induced neurotoxicity. Our data provide the first evidence that MDMA elicits a robust autophagic response in primary rat cortical neurons, as judged by an increase in autophagosome formation and protein expression of LC3B-II. The autophagy inhibitor 3-MA significantly reduced MDMA-induced autophagy activation and partially protected neurons against MDMA-induced neurite shortening and neuronal death. Either enhancement of autophagosome generation by rapamycin or impairment of autophagosome clearance by Bafilomycin A1 resulted in augmented MDMA-induced autophagosome accumulation and neurite degeneration, indicating that excessive autophagy may play a detrimental role in MDMA-triggered neurotoxicity. Furthermore, MDMA altered the phosphorylation status of AMPK and ULK1, which plays an important role in initiating autophagosome generation.

Autophagy can be classified into basal and induced modes [35]. Under basal conditions in our culture system, autophagosomes and LC3-II expression were hardly detectable in healthy neurons, although the basal level of Atg proteins, such as beclin-1 and LC3-I were highly expressed. These observations are consistent with previous observations [5]. Instead of a low level of basal autophagy, constitutive autophagy occurs in healthy neurons, and autophagic degradation is so efficient that autophagosomes cannot accumulate at detectable levels [36]. It is now well accepted that basal or low-level autophagy is cell-protective for maintenance of homeostasis and survival, while excessive or sustained autophagy can lead to self destruction or autophagic cell death, either directly or indirectly [37]. Autophagy induction and subsequent neuronal death occur in the CNS under several pathological conditions, including ischemia, trauma, glutamate-mediated excitotoxicity, and neurodegenerative protein aggregation diseases . We extended these findings by demonstrating that MDMA treatment in cultured rat cortical neurons resulted in robust autophagosome formation, concomitant with the neurodegeneration/neuronal death. Autophagic flux implies a balance between autophagosome formation and autophagic degradation. Either increased autophagosome formation in the early stage or defective degradation in the late stage can lead to accumulation of autophagic vacuoles. MDMA-induced LC3B-II accumulation could be a result of either LC3B-I to LC3B-II conversion or defects in LC3B-II degradation. In this study, 3-MA, an autophagy inhibitor at the early stage of autophagosome biogenesis, prevented MDMA-induced autophagy vacuole accumulation, suggesting MDMA may activate signaling cascades that initiate autophagosome generation. However, it cannot be completely excluded that autophagosome accumulation might be because of organelle turnover inhibition.

It is well known that MDMA induced neuron loss through apoptosis or necrosis mechanism. In the present study, we further confirm that MDMA caused neuron loss through autophagy upregulation. Extensive or dysregulated autophagy may lead to autophagic cell death, called type II programmed cell death. This mode of cell death is characterized by massive degradation of cellular contents, including essential organelles such as mitochondria, by means of complicated intracellular membrane/vesicle reorganization and lysosomal activity. Suppression of autophagy by 3-MA can block neuronal death in response to various stress conditions in the CNS such as kainate-induced excitotoxicity in rats [40], and rodent traumatic or ischemic brain injury [39], [41]. 3-MA was also found to attenuate NMDA-induced caspase 3 activation and cell death in cultured rat cerebellar granule neurons [42]. Similarly, we found that 3-MA alleviated MDMA-induced neuronal death and apoptosis, suggesting autophagic cell death involves in MDMA-induced neuron loss. However, whether other factors in MDMA-elicited autophagy and neurotoxicity, such as oxidative stress, Ca^2+^ overload, mitochondrial dysfunction or apoptosis, could contribute to autophagic cell death or require further investigation. In addition, autophagy is a double-edged sword in neuron cell survival. There is extensive cross-talk between autophagy and other forms of cell death, such as apoptosis and necrosis. Depending on cell types, environment and stimulation manners, autophagy can precede, inhibit or enhance apoptotic cell death and both autophagic and necrotic morphologies are observed in the same cell [43]. The discrepancies may be duo to the complex and diverse interactions among autophagy, apoptosis and cell death. In the present study, inhibition of autophagy by 3-MA could inhibit apoptosis induced by MDMA, suggesting involvement of autophagy-mediated apoptosis. However, we can not exclude the possibility for 3-MA direct inhibition on apoptosis. Therefore, more specific siRNA knockdown of the Atg protein (ex: Atg5 or Atg7) will be performed to elucidate the interaction between autophagy and apoptosis.

Upstream signals that regulate autophagy include class III PI3K (Vps34)/beclin 1 and AMPK/mTOR/ULK1 [13]. The specific inhibitors of autophagy (3-MA, wortmannin, and LY294002) suppress autophagy by inhibiting class III PI3K [44]. In the present study, 3-MA (but not wortmannin and LY294002) reduced MDMA-induced autophagy activation. Emerging evidence indicates that the three autophagy inhibitors can block both class I and III PI3Ks [45]. Class I and class III PI3Ks have opposing effects on autophagy—the class III pathway promotes autophagy, whereas the class I pathway inhibits autophagy by Akt/mTOR signaling [46]. This may account for the discrepancy between three inhibitors in the regulation of MDMA-induced autophagy. In addition, MDMA had no effect on protein levels of beclin-1 in this study. At present, no so-called class-specific PI3K inhibitors are available. Therefore, this raises the question of how the mechanism of 3-MA inhibition can be determined, since it involves inhibition of the pro-autophagic activity of class III PI3K. The 3-MA target(s) that mediate this effect may not act on class III PI3K directly. Previous studies have shown that 3-MA may have class III PI3K-independent effects, including mediation of Ca^2+^ flux and p38MAPK, C-Jun N-terminal kinase, and the mitochondrial permeability transition pore [47]. Interestingly, our results shown that treatment with 3-MA alone promoted neurite outgrowth, suggesting multiple pharmacological effects on biological processes in neurons. Further studies are needed to directly test these speculations.

In the present study, we employed rapamycin, Torin-1 and bafilomycin A1 to affect autophagic flux. mTOR is a central checkpoint which negatively regulates autophagy [32]. Rapamycin and Torin-1are mTOR inhibitors by inhibiting mTORC1 and both of mTORC1 and TORC2, respectively [48]. Rapamycin or Torin 1-induced autophagysome accumulation was significant augmented in neurons exposed to MDMA and subsequent neurite degeneration. However, neither rapamycin nor Torin 1 induced the formation of autophagosome, which suggesting inhibition of mTOR alone is not sufficient to induce autophagy in neurons. Similar results were also reported in previous studies, which showed that rapamycin or its analogus, everolimus weakly induced autophagy in neurons [48], [49]. In yeast, rapamycin is a potent activator of autophagy [50], whereas the situation is less clear in mammalian systems, where rapamycin alone is an inconsistent activator of autophagy and highly dependent on the cell type and treatment conditions [48]. Rapamycin frequently requires combination with other PI3K/mTOR inhibitors, such as LY294002 to enhance autophagy acitivity [51]. PI3K-Akt pathway is upstream of mTOR [52]. Therefore, we suspected that synergistic autophagy effect of MDMA plus mTOR inhibitor might dysregulated PI3K/AKt/mTOR signaling. However, detail molecular mechanism remains further investigation. Bafilomycin A1, a specific inhibitor of vacuolar H^+^ ATPase (V-ATPase), may result in an increase in autophagosomes by inhibiting fusion between autophagosomes and lysosomes [31]. However, in this study, low-dose bafilomycin A1 (1 nM) alone have less effect on autophagy in cultured cortical neuron. The results are consistent with previous study, which shown that bafilomycin A1 (≦1 nM) have no effects on LC3-II expression and autophagosome formation in cultured cerebellar neurons. High-dose Bafilomycin A1 (≧10 nM) may induced autophagy, however, we and previous study shown that this concentration of bafilomycin A1 attenuated neuronal viability [53]. Low-dose (1 nM) bafilomycin A1 did not affect neuronal viability [53] and in combination with MDMA resulted in synergistic autophagosome accumulation in our study. Together, chronicle activated or excessive autophagosome accumulation are associated with MDMA-induced neurite degeneration in cortical neurons.

Our present data revealed a marked increase in AMPK and ULK1 phosphorylation following MDMA treatment. AMPK, a heterotrimeric serine/threonine kinase, is an evolutionarily conserved cellular energy manager controlling nutrient sensing and energy homeostasis [54]. Accordingly, AMPK stimulates autophagy not only by inactivation of mTOR but also by direct phosphorylation of ULK1 [32], [55], [56]. AMPK is activated by phosphorylation of threonine residue 172, which directly increases its interaction with ULK1 and phosphorylates several serine residues of ULK1 including Ser317, Ser555, and Ser777 [14], [32]. Activation of ULK1 by AMPK can recruit Atg13 and FIP200, and trigger autophagosome formation [57]. In this study, MDMA induced phosphorylation of AMPK (Thr172) and ULK1 (Ser555), suggesting AMPK-ULK1 signaling might be involved in MDMA-induced autophagy. mTOR, another serine/threonine kinase, functions as a negative regulator of autophagy by disrupting ULK1–AMPK interaction [14], [32].

A previous study also showed that rapamycin can trigger autophagosome formation by enhancing the association of AMPK with ULK1 [55], which may account for acting synergistically with MDMA to augment autophagosome accumulation. However, the mechanism underlying MDMA-mediated AMPK-ULK1 signaling and its contribution to autophagy requires further investigation.

To conclude, we propose that MDMA neurotoxic stress triggers neuronal autophagy, which appears to be detrimental to cell survival. In other words, autophagy under the MDMA toxicity paradigm in fact contributes to the neuronal cell injury/cell death process. Abnormal accumulation of autophagic vacuoles is a critical hallmark feature of autophagic cell death. Thus, specific modulation of autophagy might be a novel neuroprotective strategy to attenuate MDMA-induced neurotoxicity.

## Supporting Information

S1 Fig **Torin-1 augmented MDMA-induced autophagy and neurodegeneration.** Cultured cortical neurons were treated with MDMA with/without 100 nM Torin-1 for 48 h. After treatment, the cells were performed double immunofluorescence with anti-LC3B and anti-MAP2 antibodies. (A) Representative double immunofluorescence and merged images using anti-LC3B and anti-MAP2 antibodies. Bar  = 30 µm. (B) Quantitation of neurite outgrowth. The total neurite length are presented as mean ± S.D., n = 3 experiments with 200–300 cells per experiment, ^#^ *P*<0.05 vs. control group; ^*^ *P*<0.05 vs. MDMA treatment.
(TIF)

## References

[1] Harris H, Rubinsztein DC (2012) Control of autophagy as a therapy for neurodegenerative disease. Nat Rev Neurol 8:108–117. 10.1038/nrneurol.2011.200 22187000

[2] Mizushima N, Levine B, Cuervo AM, Klionsky DJ (2008) Autophagy fights disease through cellular self-digestion. Nature 451:1069–1075. 18305538 10.1038/nature06639 PMC2670399

[3] Levine B, Kroemer G (2008) Autophagy in the pathogenesis of disease. Cell 132:27–42. 18191218 10.1016/j.cell.2007.12.018 PMC2696814

[4] Abounit K, Scarabelli TM, McCauley RB (2012) Autophagy in mammalian cells. World J Biol Chem 3:1–6. 22312452 10.4331/wjbc.v3.i1.1 PMC3272585

[5] Lee JA (2012) Neuronal autophagy: a housekeeper or a fighter in neuronal cell survival? Exp Neurobiol 21:1–8. 22438673 10.5607/en.2012.21.1.1 PMC3294068

[6] Yang Z, Klionsky DJ (2010) Eaten alive: a history of macroautophagy. Nat Cell Biol 12:814–822. 20811353 10.1038/ncb0910-814 PMC3616322

[7] Yang Z, Klionsky DJ (2010) Mammalian autophagy: core molecular machinery and signaling regulation. Curr Opin Cell Biol 22:124–131. 20034776 10.1016/j.ceb.2009.11.014 PMC2854249

[8] Baehrecke EH (2005) Autophagy: dual roles in life and death? Nat Rev Mol Cell Biol 6:505–510. 15928714 10.1038/nrm1666

[9] Shi R, Weng J, Zhao L, Li XM, Gao TM, et al (2012) Excessive autophagy contributes to neuron death in cerebral ischemia. CNS Neurosci Ther 18:250–260. 22449108 10.1111/j.1755-5949.2012.00295.x PMC6493486

[10] Shen HM, Codogno P (2011) Autophagic cell death: Loch Ness monster or endangered species? Autophagy 7:457–465. 21150268 10.4161/auto.7.5.14226

[11] Son JH, Shim JH, Kim KH, Ha JY, Han JY (2012) Neuronal autophagy and neurodegenerative diseases. Exp Mol Med 44:89–98. 22257884 10.3858/emm.2012.44.2.031 PMC3296817

[12] Nixon RA, Yang DS (2012) Autophagy and neuronal cell death in neurological disorders. Cold Spring Harb Perspect Biol 4. 10.1101/cshperspect.a008839 PMC3475163 22983160

[13] He C, Klionsky DJ (2009) Regulation mechanisms and signaling pathways of autophagy. Annu Rev Genet 43:67–93. 19653858 10.1146/annurev-genet-102808-114910 PMC2831538

[14] Bach M, Larance M, James DE, Ramm G (2011) The serine/threonine kinase ULK1 is a target of multiple phosphorylation events. Biochem J 440:283–291. 21819378 10.1042/BJ20101894

[15] Nakatogawa H, Suzuki K, Kamada Y, Ohsumi Y (2009) Dynamics and diversity in autophagy mechanisms: lessons from yeast. Nat Rev Mol Cell Biol 10:458–467. 19491929 10.1038/nrm2708

[16] El-Mallakh RS, Abraham HD (2007) MDMA (Ecstasy). Ann Clin Psychiatry 19:45–52. 17453661 10.1080/10401230601163592

[17] Sarkar S, Schmued L (2010) Neurotoxicity of ecstasy (MDMA): an overview. Curr Pharm Biotechnol 11:460–469. 20420572 10.2174/138920110791591490

[18] Capela JP, Carmo H, Remiao F, Bastos ML, Meisel A, et al (2009) Molecular and cellular mechanisms of ecstasy-induced neurotoxicity: an overview. Mol Neurobiol 39:210–271. 19373443 10.1007/s12035-009-8064-1

[19] Parrott AC (2002) Recreational Ecstasy/MDMA, the serotonin syndrome, and serotonergic neurotoxicity. Pharmacol Biochem Behav 71:837–844. 11888574 10.1016/s0091-3057(01)00711-0

[20] Ricaurte GA, Yuan J, McCann UD (2000) (+/-)3,4-Methylenedioxymethamphetamine ('Ecstasy')-induced serotonin neurotoxicity: studies in animals. Neuropsychobiology 42:5–10. 10867550 10.1159/000026664

[21] Schmued LC (2003) Demonstration and localization of neuronal degeneration in the rat forebrain following a single exposure to MDMA. Brain Res 974:127–133. 12742630 10.1016/s0006-8993(03)02563-0

[22] Halpin LE, Collins SA, Yamamoto BK (2014) Neurotoxicity of methamphetamine and 3,4-methylenedioxymethamphetamine. Life Sci 97:37–44. 23892199 10.1016/j.lfs.2013.07.014 PMC3870191

[23] Soleimani Asl S, Farhadi MH, Moosavizadeh K, Samadi Kuchak Saraei A, Soleimani M, et al (2012) Evaluation of Bcl-2 Family Gene Expression in Hippocampus of 3, 4-methylenedioxymethamphetamine Treated Rats. Cell J 13:275–280. 23508090 PMC3584478

[24] Chae M, Rhee GS, Jang IS, Kim K, Lee JH, et al (2009) ATG5 expression induced by MDMA (ecstasy), interferes with neuronal differentiation of neuroblastoma cells. Mol Cells 27:571–575. 19466606 10.1007/s10059-009-0075-2

[25] Smits A, Kato M, Westermark B, Nister M, Heldin CH, et al (1991) Neurotrophic activity of platelet-derived growth factor (PDGF): Rat neuronal cells possess functional PDGF beta-type receptors and respond to PDGF. Proc Natl Acad Sci U S A 88:8159–8163. 1654560 10.1073/pnas.88.18.8159 PMC52466

[26] Munafo DB, Colombo MI (2001) A novel assay to study autophagy: regulation of autophagosome vacuole size by amino acid deprivation. J Cell Sci 114:3619–3629. 11707514 10.1242/jcs.114.20.3619

[27] Popko J, Fernandes A, Brites D, Lanier LM (2009) Automated analysis of NeuronJ tracing data. Cytometry A 75:371–376. 18937344 10.1002/cyto.a.20660 PMC2661008

[28] Kabeya Y, Mizushima N, Ueno T, Yamamoto A, Kirisako T, et al (2000) LC3, a mammalian homologue of yeast Apg8p, is localized in autophagosome membranes after processing. EMBO J 19:5720–5728. 11060023 10.1093/emboj/19.21.5720 PMC305793

[29] Biederbick A, Kern HF, Elsasser HP (1995) Monodansylcadaverine (MDC) is a specific in vivo marker for autophagic vacuoles. Eur J Cell Biol 66:3–14. 7750517

[30] Seglen PO, Gordon PB (1982) 3-Methyladenine: specific inhibitor of autophagic/lysosomal protein degradation in isolated rat hepatocytes. Proc Natl Acad Sci U S A 79:1889–1892. 6952238 10.1073/pnas.79.6.1889 PMC346086

[31] Yamamoto A, Tagawa Y, Yoshimori T, Moriyama Y, Masaki R, et al (1998) Bafilomycin A1 prevents maturation of autophagic vacuoles by inhibiting fusion between autophagosomes and lysosomes in rat hepatoma cell line, H-4-II-E cells. Cell Struct Funct 23:33–42. 9639028 10.1247/csf.23.33

[32] Kim J, Kundu M, Viollet B, Guan KL (2011) AMPK and mTOR regulate autophagy through direct phosphorylation of Ulk1. Nat Cell Biol 13:132–141. 21258367 10.1038/ncb2152 PMC3987946

[33] Egan DF, Shackelford DB, Mihaylova MM, Gelino S, Kohnz RA, et al (2011) Phosphorylation of ULK1 (hATG1) by AMP-activated protein kinase connects energy sensing to mitophagy. Science 331:456–461. 21205641 10.1126/science.1196371 PMC3030664

[34] Halpin LE, Collins SA, Yamamoto BK (2013) Neurotoxicity of methamphetamine and 3,4-methylenedioxymethamphetamine. Life Sci 97:37–44. 23892199 10.1016/j.lfs.2013.07.014 PMC3870191

[35] Mizushima N (2005) The pleiotropic role of autophagy: from protein metabolism to bactericide. Cell Death Differ 12 Suppl 2: 1535–1541. 16247501 10.1038/sj.cdd.4401728

[36] Boland B, Kumar A, Lee S, Platt FM, Wegiel J, et al (2008) Autophagy induction and autophagosome clearance in neurons: relationship to autophagic pathology in Alzheimer's disease. J Neurosci 28:6926–6937. 18596167 10.1523/JNEUROSCI.0800-08.2008 PMC2676733

[37] Kroemer G, Levine B (2008) Autophagic cell death: the story of a misnomer. Nat Rev Mol Cell Biol 9:1004–1010. 18971948 10.1038/nrm2527 PMC2727358

[38] Perez-Carrion MD, Perez-Martinez FC, Merino S, Sanchez-Verdu P, Martinez-Hernandez J, et al (2012) Dendrimer-mediated siRNA delivery knocks down Beclin 1 and potentiates NMDA-mediated toxicity in rat cortical neurons. J Neurochem 120:259–268. 22035151 10.1111/j.1471-4159.2011.07556.x

[39] Luo CL, Li BX, Li QQ, Chen XP, Sun YX, et al (2011) Autophagy is involved in traumatic brain injury-induced cell death and contributes to functional outcome deficits in mice. Neuroscience 184:54–63. 21463664 10.1016/j.neuroscience.2011.03.021

[40] Wang Y, Han R, Liang ZQ, Wu JC, Zhang XD, et al (2008) An autophagic mechanism is involved in apoptotic death of rat striatal neurons induced by the non-N-methyl-D-aspartate receptor agonist kainic acid. Autophagy 4:214–226. 18094625 10.4161/auto.5369

[41] Wang JY, Xia Q, Chu KT, Pan J, Sun LN, et al (2011) Severe global cerebral ischemia-induced programmed necrosis of hippocampal CA1 neurons in rat is prevented by 3-methyladenine: a widely used inhibitor of autophagy. J Neuropathol Exp Neurol 70:314–322. 21412169 10.1097/NEN.0b013e31821352bd

[42] Sadasivan S, Zhang Z, Larner SF, Liu MC, Zheng W, et al (2010) Acute NMDA toxicity in cultured rat cerebellar granule neurons is accompanied by autophagy induction and late onset autophagic cell death phenotype. BMC Neurosci 11:21. 20167092 10.1186/1471-2202-11-21 PMC2836363

[43] Levine B, Yuan J (2005) Autophagy in cell death: an innocent convict? J Clin Invest 115:2679–2688. 16200202 10.1172/JCI26390 PMC1236698

[44] Takatsuka C, Inoue Y, Matsuoka K, Moriyasu Y (2004) 3-methyladenine inhibits autophagy in tobacco culture cells under sucrose starvation conditions. Plant Cell Physiol 45:265–274. 15047874 10.1093/pcp/pch031

[45] Wu YT, Tan HL, Shui G, Bauvy C, Huang Q, et al (2010) Dual role of 3-methyladenine in modulation of autophagy via different temporal patterns of inhibition on class I and III phosphoinositide 3-kinase. J Biol Chem 285:10850–10861. 20123989 10.1074/jbc.M109.080796 PMC2856291

[46] Petiot A, Ogier-Denis E, Blommaart EF, Meijer AJ, Codogno P (2000) Distinct classes of phosphatidylinositol 3'-kinases are involved in signaling pathways that control macroautophagy in HT-29 cells. J Biol Chem 275:992–998. 10625637 10.1074/jbc.275.2.992

[47] Scarlatti F, Granata R, Meijer AJ, Codogno P (2009) Does autophagy have a license to kill mammalian cells? Cell Death Differ 16:12–20. 18600232 10.1038/cdd.2008.101

[48] Thoreen CC, Kang SA, Chang JW, Liu Q, Zhang J, et al (2009) An ATP-competitive mammalian target of rapamycin inhibitor reveals rapamycin-resistant functions of mTORC1. J Biol Chem 284:8023–8032. 19150980 10.1074/jbc.M900301200 PMC2658096

[49] Tsvetkov AS, Miller J, Arrasate M, Wong JS, Pleiss MA, et al (2010) A small-molecule scaffold induces autophagy in primary neurons and protects against toxicity in a Huntington disease model. Proc Natl Acad Sci U S A 107:16982–16987. 20833817 10.1073/pnas.1004498107 PMC2947884

[50] Noda T, Ohsumi Y (1998) Tor, a phosphatidylinositol kinase homologue, controls autophagy in yeast. J Biol Chem 273:3963–3966. 9461583 10.1074/jbc.273.7.3963

[51] Takeuchi H, Kondo Y, Fujiwara K, Kanzawa T, Aoki H, et al (2005) Synergistic augmentation of rapamycin-induced autophagy in malignant glioma cells by phosphatidylinositol 3-kinase/protein kinase B inhibitors. Cancer Res 65:3336–3346. 15833867 10.1158/0008-5472.CAN-04-3640

[52] Heras-Sandoval D, Perez-Rojas JM, Hernandez-Damian J, Pedraza-Chaverri J (2014) The role of PI3K/AKT/mTOR pathway in the modulation of autophagy and the clearance of protein aggregates in neurodegeneration. Cell Signal 26:2694–2701. 25173700 10.1016/j.cellsig.2014.08.019

[53] Shacka JJ, Klocke BJ, Shibata M, Uchiyama Y, Datta G, et al (2006) Bafilomycin A1 inhibits chloroquine-induced death of cerebellar granule neurons. Mol Pharmacol 69:1125–1136. 16391239 10.1124/mol.105.018408

[54] Ramamurthy S, Ronnett G (2012) AMP-Activated Protein Kinase (AMPK) and Energy-Sensing in the Brain. Exp Neurobiol 21:52–60. 22792025 10.5607/en.2012.21.2.52 PMC3381212

[55] Lee JW, Park S, Takahashi Y, Wang HG (2010) The association of AMPK with ULK1 regulates autophagy. PLoS One 5:e15394. 21072212 10.1371/journal.pone.0015394 PMC2972217

[56] Alers S, Loffler AS, Wesselborg S, Stork B (2012) Role of AMPK-mTOR-Ulk1/2 in the regulation of autophagy: cross talk, shortcuts, and feedbacks. Mol Cell Biol 32:2–11. 22025673 10.1128/MCB.06159-11 PMC3255710

[57] Jung CH, Jun CB, Ro SH, Kim YM, Otto NM, et al (2009) ULK-Atg13-FIP200 complexes mediate mTOR signaling to the autophagy machinery. Mol Biol Cell 20:1992–2003. 19225151 10.1091/mbc.E08-12-1249 PMC2663920

