# Supplementary figures and images for: Autophagy Activation Is Involved in 3,4-Methylenedioxymethamphetamine (‘Ecstasy’)—Induced Neurotoxicity in Cultured Cortical Neurons
Source: PLoS One. 2014 Dec 31;9(12):e116565. doi: 10.1371/journal.pone.0116565 (PMC4281065; doi:10.1371/journal.pone.0116565)

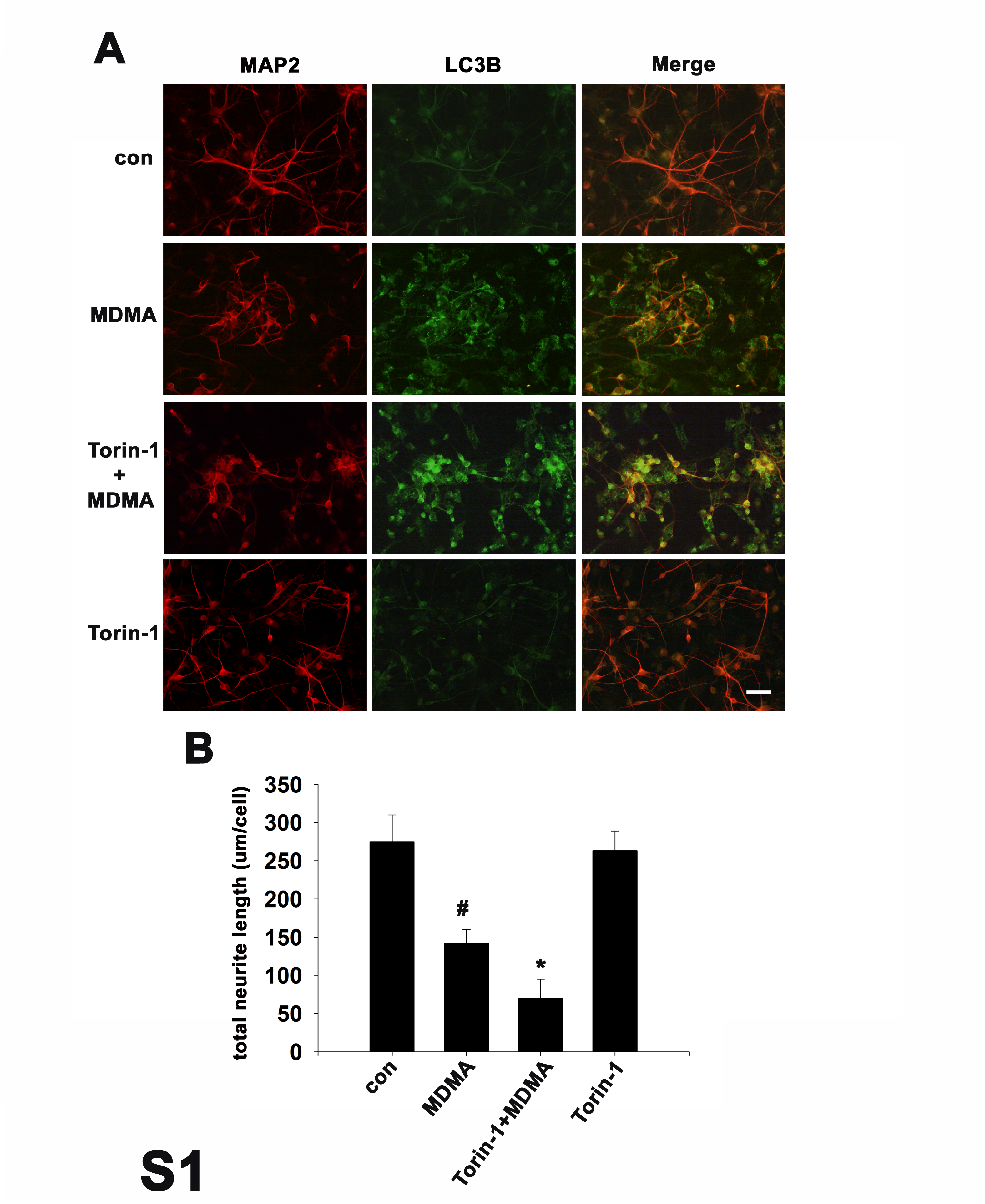

Supplement: S1 Fig — Torin-1 augmented MDMA-induced autophagy and neurodegeneration. Cultured cortical neurons were treated with MDMA with/without 100 nM Torin-1 for 48 h. After treatment, the cells were performed double immunofluorescence with anti-LC3B and anti-MAP2 antibodies. (A) Representative double immunofluorescence and merged images using anti-LC3B and anti-MAP2 antibodies. Bar = 30 µm. (B) Quantitation of neurite outgrowth. The total neurite length are presented as mean ± S.D., n = 3 experiments with 200–300 cells per experiment, # P<0.05 vs. control group; * P<0.05 vs. MDMA treatment. (TIF) [file pone.0116565.s001.tif]
